# The effect of a hospital liaison psychiatry service on inpatient lengths of stay: interrupted time series analysis using routinely collected NHS hospital episode statistics

**DOI:** 10.1186/s12888-020-2441-8

**Published:** 2020-01-28

**Authors:** Allan House, Robert West, Chris Smith, Sandy Tubeuf, Else Guthrie, Peter Trigwell

**Affiliations:** 1 Leeds Institute of Health Sciences, University of Leeds, Leeds, UK; 2 Institute of Health and Society, Université catholique de Louvain, Louvain, Belgium; 3 National Inpatient Centre for Psychological Medicine, Leeds and York Partnership NHS Foundation Trust, Leeds, UK

## Abstract

**Background:**

The purpose of the study was to determine whether establishment of a specific liaison psychiatry service designed to offer a rapid response with facilitated hospital discharge led to reduced acute hospital length of inpatient stay.

**Methods:**

We used interrupted time series based upon routine NHS data from secondary care service in two acute general hospitals, for all adult (16+ years) inpatient admissions (114,029 inpatient spells representing 70,575 individual patients) over 3 years.

**Results:**

Length of stay reduced over time in both hospitals. Against a background of falling length of stay across the study period, there was no discernible effect of the rapid access/early discharge liaison service on length of stay, either as a step change or linear decline. This finding held for all patients and for those over 65 years and those discharged with a mental health diagnosis.

**Conclusions:**

Using routine NHS data for a whole hospital it was not possible to replicate a previous report that a rapid access liaison psychiatry service for inpatients produces substantial reductions in length of stay, and commissioners of services should be cautious of claims to the contrary. Further research to determine if there is an effect for sub-groups will require major improvements in the way co-morbid mental disorders are coded in NHS practice.

## Background

Liaison psychiatry is the sub-specialty of psychiatry concerned with clinical practice, teaching and research in physical healthcare settings. A 2015 survey of all acute general hospitals in England reported that 168 out of the 179 acute hospitals with an emergency department had some form of liaison psychiatry service [1]. Further expansion is planned, with a national target that at least 50% of all acute hospitals in England will have liaison services staffed to key commissioning standards by 2020/21 [2, 3], 70% by 2023/24 and 100% in the long term [4].

The rationale for this recent investment in liaison psychiatry in the UK has been focused on two issues – the need to provide equitable access to emergency care for all patients regardless of whether their problems are diagnosed as physical or mental, and the suggestion that cost savings might result from the service. The suggestion that financial savings from timely psychiatric intervention are substantial enough that they can pay for the liaison psychiatry service, the so-called “cost-offset” effect, is not a new one [5, 6]. Although evidence for this idea has not been overwhelming, [7] it has attracted much recent interest in the UK following the publication of a report from one English hospital, which reported on a so-called Rapid Access Intervention and Discharge service, which at the time used the acronym RAID.^1^ The service focused on rapid response to inpatient ward referrals with assessment and care plans concentrated on achieving early discharge from hospital, and results were reported as showing reductions in average inpatient lengths of stay in the target population of up to 5 days, mainly among patients not seen by the service – described as an indirect effect. A widely circulated estimate from the study is that for each £1 invested in liaison psychiatry services, £4 could be saved by the resultant reduction in medical bed usage [8, 9].

There were however important weaknesses in the original evaluation, which was based upon a before-and-after design using short evaluation epochs; case-by-case matching led to small numbers in the comparison groups; confounding by indication remained a possibility, and there was little contextual information to aid interpretation of the findings. Further evaluations of liaison services in Sheffield [10] Bristol [11], Newcastle [12] and London Hospitals [13] have also suggested savings, and although the methods of evaluation have varied they have all involved comparisons of retrospective data in the year before and after service introduction.

Because results from one centre cannot routinely be applied to other centres where context may exert different influences on outcomes, it is important for commissioners and providers of services to be able to evaluate for themselves the effects of local liaison psychiatry services. For the efficiency of such evaluations, it would be helpful to know if routinely collected hospital data can be used to assess the performance of liaison services, including performance against metrics such as lengths of stay in the hospital where they are located. We therefore undertake a study of changes in length of hospital stay over 3 years in two general hospitals in the same city in England, in only one of which (City Hospital, Birmingham) the liaison psychiatry service described at that time as RAID had been introduced at the mid-point of the study period. Our main objective is to determine whether it is possible to discern an effect of the introduction of the liaison service on lengths of stay using routine NHS data relating to hospital episodes.

### Methods

Ethical approval for the study was obtained from the School of Medicine Research Ethics Committee, University of Leeds (SoMREC/13/059).

### Setting

Two acute general hospitals with emergency departments. The hospitals are in the same city in the Midlands of England. In Queen Elizabeth II Hospital (hereafter QE), Birmingham, there is no liaison psychiatry service during the study period (Quarter 2, 2007 to Quarter 3, 2011). In City Hospital, Birmingham (hereafter City), the liaison psychiatry service known then as RAID was introduced at the end of Quarter 4, 2009.

### Design

Quasi-experimental design using interrupted time series analysis. We follow the Strengthening the Reporting of Observational Studies in Epidemiology (STROBE) guidelines [14] for the reporting of observational studies.

### Measures

We obtained data relating to inpatient admissions from the Hospital Episode Statistics (HES) database for the period Quarter 2, 2007 – Quarter 3, 2011.

- admission type- whether emergency (unscheduled) or planned (elective).
- Length of inpatient spell (days)
- 28 day readmissions
- The whole sample included all patients aged 16 + years at discharge,
- Mental health diagnosis coded (primary or secondary diagnosis) at time of discharge.

HES data are derived from data routinely provided by all NHS inpatient facilities. They are subject to quality checks by NHS Digital, the recognized data controller and thereafter published in a standard format.

### Analysis

Length of stay (LOS) data have a large right skew. We made the assumption that distribution of LOS is log normal [15], and therefore that Log (LOS) is normally distributed.

We built a regression model that permitted both a step change at the introduction of the service then called RAID and an overall downward linear slope which could change at the date of introduction. Length of stay was studied for the two years before and one year after the date at which a liaison psychiatry service was introduced at the City Hospital. We chose the shorter period after introduction because a major change underway in the city at the time was that a new hospital was being built, opening in Quarter 3, 2010 and we expected this to make interpretation of LOS data for the other hospitals too complicated.

We used essentially the same method to model 28 day readmission, applying logistic regression to the data.

For both outcomes (LOS, 28 day readmission) modelling a step change used change as a constant term, a trend as a linear term over time. We sought to determine which model provided the best fit to the data.

Both admission type and prevalence of discharge with a mental health diagnosis change over time. It is possible that other aspects of case mix change over time – due to changes in NHS context, seasonal effects, and/or the impact of case ascertainment. We therefore modelled LOS including co-factors for age group, admission type and the presence of delirium/dementia as fixed effects in the model. We chose dementia for two reasons – because we needed to reduce the number of diagnostic categories to allow efficient modelling, and because the previous reports cited above suggested that it was older people with mental health problems in whom the greatest reductions in LOS were seen.

We fitted the regression for the full 3 years for each hospital (two years pre, one year post).

We fitted with a multilevel model, which is better than treating admissions as independent: it is best practice to treat admissions as being nested within patients.

As a sensitivity analysis we modelled the data assuming Length of stay minus one day (LOS-1) has a negative binomial distribution.

## Results

Data were obtained for 114,029 inpatient spells during the study period, representing 70,575 individual patients. At City, there were 72,929 admissions for 48,229 patients, at QE 69,515 admissions for 45,104 patients.

The characteristics of patients for the two hospitals are presented in Tables 1 and 2, which give data for the 9-month period before the date at which a liaison psychiatry service was introduced at the City Hospital. Admissions at the City Hospital were more likely to be older, to receive more mental health diagnoses and to be far more often non-elective. Mental health diagnoses at City Hospital increased in the 9 months after introduction of the liaison psychiatry service. LOS throughout the period was shorter at City Hospital.

**Table 1:** Admissions data at City Hospital. Comparisons are by admission rather than patient

Characteristics	Categories	Before	After	*p*-value
Number of admissions/stays		15,916	16,862	
Number of patients		12,892	13,618	
LOS (median IQR)		2 [1, 6]	2 [1,6]	0.024
Log LOS (mean (sd))		1.06 (1.12)	1.04 (1.10)	0.018
Age (mean (sd))		55.92 (21.24)	55.75 (21.19)	0.455
Age	16–40 (n (%))	4246 (26.7%)	4465 (26.5%)	0.345
	40–50 (n (%))	2131 (13.4%)	2316 (13.7%)	
	50–60 (n (%))	2004 (12.6%)	2141 (12.7%)	
	60–70 (n (%))	2240 (14.1%)	2481 (14.7%)	
	70–80 (n (%))	2859 (18.0%)	2915 (17.3%)	
	80–109 (n (%))	2436 (15.3%)	2544 (15.1%)	
Admission type	Elective (n (%))	4278 (26.9%)	4251 (25.2%)	0.001
	Other (n (%))	11,638 (73.1%)	12,611 (74.8%)	
Mental health	None (n (%))	13,555 (85.2%)	14,159 (84.0%)	0.022
	Alcohol (n (%))	983 (6.2%)	1155 (6.8%)	
	Dementia/Delirium (n (%))	211 (1.3%)	226 (1.3%)	
	Depression (n (%))	642 (4.0%)	760 (4.5%)	
	Other (n (%))	525 (3.3%)	562 (6.8%)	
Readmission within 28 days	No (n (%))	13,581 (85.3%)	14,341 (85.0%)	0.485
	Yes (n (%))	2335 (14.7%)	2521 (15.0%)	

*sd* standard deviation

*IQR* interquartile range

*LOS* length of stay

**Table 2:** Admissions data at Queen Elizabeth Hospital. Comparisons are by admission rather than patient

Characteristic	Level	Before	After	p-value
Number of admissions/stays		15,823	15,018	
Number of patients		12,257	11,614	
LOS (median IQR)		3 [1, 7]	3 [1,7]	0.424
Log LOS (mean (sd))		1.25 (1.05)	1.26 (1.06)	0.291
Age (mean (sd))		53.75 (18.49)	54.52 (18.43)	< 0.001
Age	16–40 (n (%))	3821 (24.1%)	3407 (22.7%)	0.057
	40–50 (n (%))	2348 (14.8%)	2245 (14.9%)	
	50–60 (n (%))	2843 (18.0%)	2697 (18.0%)	
	60–70 (n (%))	3222 (20.4%)	3099 (20.6%)	
	70–80 (n (%))	2524 (16.0%)	2481 (16.5%)	
	80–109 (n (%))	1074 (6.8%)	1089 (7.3%)	
Admission type	Elective (n (%))	8916 (56.3%)	8330 (55.5%)	0.112
	Other (n (%))	6907 (43.7%)	6688 (44.5%)	
Mental health	None (n (%))	15,167 (95.9%)	14,395 (95.9%)	0.433
	Alcohol (n (%))	185 (1.2%)	169 (1.1%)	
	Dementia/Delirium (n (%))	24 (0.2%)	28 (0.2%)	
	Depression (n (%))	303 (1.9%)	312 (2.1%)	
	Other (n (%))	144 (0.9%)	114 (0.8%)	
Readmission within 28 days	No (n (%))	12,814 (81.0%)	12,083 (80.5%)	0.247
	Yes (n (%))	3009 (19.0%)	2935 (19.5%)	

*sd* standard deviation

*IQR* interquartile range

*LOS* length of stay

**Fig. 1:**
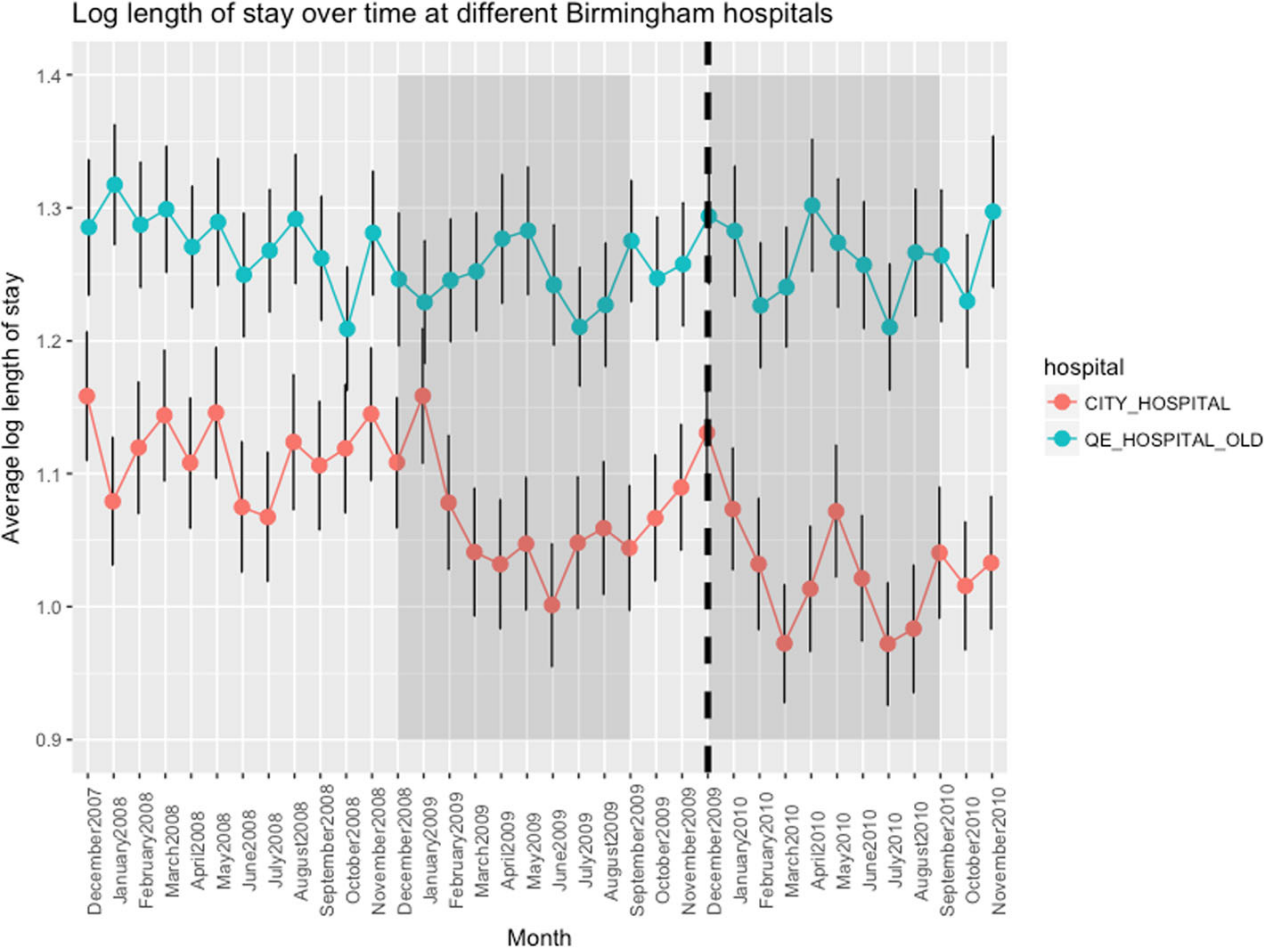
The use of the RAID acronym has been the subject of legal proceedings - APT Training & Consultancy Ltd & Anor v Birmingham & Solihull Mental Health NHS Trust [2019] EWHC 19 (IPEC) (09 January 2019). The finding was that use of the acronym to describe various liaison psychiatry activities represents infringement of the Registered Trade Marks. In this paper we use the acronym for the avoidance of doubt to refer to reports of the original study published using the acronym, before the judgement

Data for length of stay for each quarter of the study period are presented graphically in Fig. 1. Shaded areas show the periods included in the model, before and after introduction of the City Hospital liaison psychiatry service. The dashed vertical is at 1 Dec 2009 when the service was introduced. For the purposes of visualisation the Figure uses data averaged over patients admitted over each month listed on the abscissa, although the analysis made use of the continuous/daily specification of time from HES.

Results for the two models fitted to data from each of the two hospitals are shown in Tables 3 and 4. As expected, LOS increased with age and mental health diagnosis. There is a general decrease in log-LOS over time, which is statistically highly significant. There is little evidence of either a step change or a change in slope of the overall trend from the time of introduction of a liaison psychiatry service.

**Table 3:** City Hospital - Ordinary Least Square estimated coefficients with individual and episode fixed effects, based upon 72,929 admissions for 48,229 patients

	Estimate	Standard error	t-value	p-value
Intercept	0.616	0.012		
MH - none	Reference			
Alcohol	0.156	0.017	9.11	< 0.001
Delirium	0.995	0.175	5.70	< 0.001
Dementia	0.958	0.035	18.66	< 0.001
Depression	0.208	0.019	10.72	< 0.001
Multiple MH	0.066	0.030	2.18	0.035
Psychosis	0.322	0.040	7.97	< 0.001
Age 16–40	Reference			
40–50	0.195	0.014	14.25	< 0.001
50–60	0.332	0.014	23.59	< 0.001
60–70	0.511	0.014	37.68	< 0.001
70–80	0.799	0.013	62.10	< 0.001
80–109	1.125	0.014	82.39	< 0.001
Time per year	−0.044	0.008	−5.44	< 0.001
Step change	0.045	0.057	0.79	0.430
Change of slope	−0.010	0.024	−0.42	0.674

Random intercept – summarised by intra class coefficient (ICC) = 0.209

*MH* mental health

**Table 4:** Queen Elizabeth Hospital - Ordinary Least Square estimated coefficients with individual and episode fixed effects, based upon 69,515 admissions for 45,104 patients

	Estimate	Standard error	t-value	p-value
Intercept	1.006	0.013		
MH - none	Reference			
Alcohol	0.356	0.037	9.43	< 0.001
Delirium	1.128	0.224	5.02	< 0.001
Dementia	0.636	0.110	5.79	< 0.001
Depression	0.203	0.030	6.75	< 0.001
Multiple MH	0.513	0.083	0.616	< 0.001
Psychosis	0.362	0.092	2.10	0.036
Age 16–40	Reference			
40–50	0.172	0.014	11.85	< 0.001
50–60	0.290	0.014	21.08	< 0.001
60–70	0.362	0.013	27.35	< 0.001
70–80	0.444	0.014	31.57	< 0.001
80–109	0.474	0.019	25.84	< 0.001
Time per year	−0.026	0.008	−3.07	0.002
Step change	0.030	0.065	0.45	0.653
Change of slope	0.001	0.027	0.02	0.984

Random intercept – summarised by ICC = 0.193

*MH* mental health

In a sensitivity analysis, the above models were fitted assuming that LOS-1 follows a negative binomial distribution. With a random intercept, the full model fails to converge numerically. With a step change and a change in slope fitted in separate models these terms are not statistically significant, the fixed effects are very similar, and the interpretation of the results is exactly the same.

The fitted models are consistent with an overall decrease in hospital LOS over time and this does not appear to be specifically due to the introduction of the liaison psychiatry service. The general decline in LOS is greater for City Hospital than for the Queen Elizabeth Hospital over the study period.

There was no evidence in either hospital of an increase in the 28-day readmission rates during the study period.

## Discussion

### Main findings

We found that the model of LOS data that provided the best fit is gradual reduction in LOS with no step following the introduction of the Birmingham City RAID service. There is a time-year effect in the model with a strong age effect (for patients aged 65 years and above) in addition to which a discharge diagnosis of dementia or delirium added little to the model. In none of our models was there an apparent effect of the service. This gradual decline in lengths of stay was apparent in both the hospitals we studied, and although it was slightly faster in City Hospital that trajectory was apparent before the new service started.

### Main strengths and weaknesses

The main strengths and limitations of our study reside in the use of the HES dataset, which provides a comprehensive unbiased record of all inpatient admissions. In our analysis we took account of clustering of admissions within patients by the inclusion of a random intercept for patients. This very significantly improved the model fit and consequently the reliability of our findings.

Mental health coding is known to be unreliable in routine data, [16] especially for the commoner non-psychotic disorders, and here it was unreliable as judged by the low prevalence of a number of the key mental disorders coded in the HES dataset. The explanation goes beyond liaison services because most diagnoses are applied by general hospital staff; attention to diagnosis and coding can lead to quality improvement for those cases seen by the liaison team but is likely to remain a limitation of routine data for the foreseeable future.

Whether as a result of changes in coding practice or as a result of genuine changes in case-mix, there were changes in the number of mental health codes assigned after the implementation of the RAID service and this raises a question about the reliability of interrupted time series for analysing changes in LOS for the subgroup with a mental health diagnosis, which depends upon stability in the denominator of interest.

The study examined data from two hospitals in one city in England, so the overall results may not be generalizable to other hospitals, which will have different local pressures, different types of liaison services and different overall LOS. The methods are transferable and suggest that any robust evaluation of liaison services must take account of the general trend in hospital LOS in the particular hospital being evaluated.

### Comparison with other studies

An older cost-offset literature suggested savings as a consequence of reducing hospital lengths of stay. These studies were however conducted in a very different era (the 1980s) and not in the UK. A more recent study from the USA that examined changes in LOS over a 10- year period also suggested that the LOS of stay of patients seen by a hospital liaison team fell more sharply than the overall length of stay, over the same period of time [17]. The health care systems in the USA and UK are however very different. In recent years, in England, there has been severe pressure on hospital costs and continuing attempts to realise early hospital discharge – aided on occasions by technological advances in medicine but also by an increased emphasis on community treatment for many disorders. Recent UK attempts at replication of the findings from Birmingham using before-and-after designs have suggested savings, but share many of the original study’s methodological weaknesses.

Our own study is not a direct replication of the Birmingham study, which involved a before-and-after study only of patients who received a mental health diagnosis as part of their acute hospital admission. We were interested in the broader question, of relevance to service planners and providers, of whether routine NHS data could be used to establish whole-hospital effects of liaison psychiatry services on lengths of stay in an acute hospital.

### Implications

When using a model that took account of longer-term trends in LOS rather than one that simply looked for a before-and after change, we found no strong evidence for an effect of the RAID service upon hospital-wide LOS of a magnitude likely to lead to cost-savings of the order previously reported. This suggests that the main effect on LOS lies with factors other than liaison psychiatry – as suggested both by the fall in LOS in the hospital we used for comparison and in falling LOS for older adults with a mental health diagnosis, which were observed before the introduction of the service. One plausible interpretation is therefore that the introduction of the liaison psychiatry service was a sign of a desire to reduce LOS further, for example for economic or social reasons, rather than a cause of such a reduction. An alternative explanation is that there is indeed a liaison psychiatry effect on LOS, which appears as a continuation of the fall in LOS, which would otherwise have reached a plateau due to the limitations of continuing early discharges.

It is perhaps unrealistic to suggest that liaison services have major impacts upon patients who are not even assessed or treated by liaison teams, or that reductions in LOS of patients assessed by liaison teams will have an impact on overall hospital figures; the referral rate to liaison services has been estimated at between 0.4 to 5.8% of all hospital in-patients [18–21]. The small percentage of patients who are referred to liaison services, however, have longer than average hospital LOS, so there is some potential for a modest cost offset, but this direct patient effect has not been studied adequately in a UK setting. It may be that LOS can be reduced in selected sub-groups of patients, but if so the numbers are too small to exert an impact on overall hospital LOS. Proactive liaison models are being developed, where a liaison psychiatrist reviews all medical admissions, with a medical team, then decides with which patients to intervene. A preliminary evaluation suggests a positive impact on LOS [22] but further robust evaluation is required and at present this way of working does not reflect routine clinical practice in the NHS.

There are of course other reasons than reduction in LOS to support liaison psychiatry services – the need for healthcare for complex cases of mental and physical co-morbidity, medically unexplained syndromes or urgent psychiatric presentations in ED. Ward work that doesn’t reduce LOS can be important if it brings quality of life benefits or improves disease-specific clinical outcomes regardless of effects on length of stay.

Future work in this area will be improved by more accurate methods of defining case-mix. Routine general hospital data are unlikely to be a source of relevant data in the foreseeable future and one possibility is to obtain information from other sources via record linkage – especially primary care and (for those seen in the liaison service) mental health service records.

There are implications of our work for service commissioning. Recent NICE Guidelines published last year recognised the potential serious limitations of the ‘RAID’ evaluation [23] Carrying out this sort of analysis in routine NHS practice is difficult – requiring expertise in data science and statistical modelling that is not readily available outside academic centres. Most liaison services aren’t starting from scratch and interrupted time series is not a feasible method for picking up effects of smaller changes in service staffing. Service commissioners and providers need to be aware of this difficulty – and yet we are aware of instances of unrealistic requests for evidence of changes in costs based upon LOS reduction as a result of investment in liaison services.

#### Acknowlegements

Not applicable.

#### Ethical approval and consent

Ethical permission was obtained from School of Medicine Research Ethics Committee, University of Leeds SoMREC/13/059.

## Data Availability

Data used in this study were derived from inpatient admissions contained within the Hospital Episode Statistics (HES) database. HES is controlled by NHS Digital. Access to HES for this study is subject to a Data Sharing Agreement between the University of Leeds and NHS Digital.

## Abbreviations

HES: Hospital Episode Statistics
ICC: Intra-class coefficient
IQR: Interquartile range
LOS: Length of Stay
MH: Mental Health
NHS: National Health Service
RAID: Rapid Access Intervention and Discharge Services
SD: Standard deviation
STROBE: Strengthening the Reporting of Observational Studies in Epidemiology

## Footnotes

1 The use of the RAID acronym has been the subject of legal proceedings - APT Training & Consultancy Ltd. & Anor v Birmingham & Solihull Mental Health NHS Trust [2019] EWHC 19 (IPEC) (09 January 2019). The finding was that use of the acronym to describe various liaison psychiatry activities represents infringement of the Registered Trade Marks. In this paper we use the acronym for the avoidance of doubt to refer to reports of the original study published using the acronym, before the judgement.
